# Prognostic value of long non-coding RNA PVT1 as a novel biomarker in various cancers: a meta-analysis

**DOI:** 10.18632/oncotarget.22830

**Published:** 2017-12-01

**Authors:** Shikai Zhu, Ping Shuai, Chong Yang, Yun Zhang, Shan Zhong, Xingchao Liu, Kai Chen, Qin Ran, Hongji Yang, Yu Zhou

**Affiliations:** ^1^ Organ Transplant Center, Hospital of University of Electronic Science and Technology of China and Sichuan Provincial People's Hospital, Chengdu 610072, Sichuan, P.R.China; ^2^ Sichuan Provincial Key Laboratory for Human Disease Gene Study and Institute of Laboratory Medicine, Hospital of University of Electronic Science and Technology of China and Sichuan Provincial People's Hospital, Chengdu 610072, Sichuan, P.R.China; ^3^ School of Medicine, University of Electronic Science and Technology of China, Chengdu 610054, Sichuan, P.R.China

**Keywords:** long non-coding RNA, PVT1, cancer, prognosis, lymph node metastasis

## Abstract

**Background:**

Plasmacytoma variant translocation 1 (PVT1) has recently been reported to be aberrantly expressed and serves as a prognostic biomarker in many types of cancers. However, its prognostic significance remains controversial. Here, we conducted a meta-analysis to investigate the prognostic value of PVT1 expression in cancers.

**Results:**

A total of 2109 patients from 20 studies were included. The results showed that elevated PVT1 expression predicted a poor outcome for overall survival (OS) in nine types of cancers (HR = 1.40, 95% CI: 1.21–1.59). Subgroup analysis indicated that there was a significant association between PVT1 overexpression and poor OS of patients with gastric cancer, gynecology cancer and lung cancer. Furthermore, we also found a negative significant relationship between PVT1 expression and disease-free survival (HR = 1.83, 95% CI: 1.39–2.27), progression-free survival (HR = 1.63, 95% CI: 1.34–1.93) and recurrence-free survival (HR = 1.74, 95% CI: 1.01–2.47). In addition, the level of PVT1 expression was positively related to tumor size, TNM stage, lymph node metastasis and distant metastases.

**Materials and Methods:**

A systematic search was performed through the PubMed, EMBASE, Web of Science, Ovid and Cochrane library databases for eligible studies on prognostic value of PVT1 in cancers from inception up to June, 2017. The pooled hazard ratios (HRs) or odds ratios (ORs) with 95% confidence intervals (CIs) were used to evaluate the association between PVT1 expression and clinical outcomes.

**Conclusions:**

PVT1 expression positively related to tumor size, TNM stages, lymph node metastasis and distant metastases, and served as a prognostic biomarker in different types of cancers.

## INTRODUCTION

Long noncoding RNAs (lncRNAs) are evolutionarily conserved non-protein coding RNAs that are longer than 200 nucleotides in length [1]. It has been reported that lncRNAs regulate gene expression through diverse molecular processes, including transcriptional and posttranscriptional processing, chromatin modification and epigenetics, genomic imprinting, protein activity modulation and protein localization [2]. Emerging evidence has indicated that dysregulation of lncRNAs is often associated with a variety of human diseases including cancer [3, 4]. Importantly, lncRNAs could play important regulators in various cancer-related processes such as modulation of apoptosis and proliferation, drug resistance and the process of epithelial-mesenchymal transition [5]. Therefore, lncRNAs might act as potential valuable biomarkers for cancer diagnosis or potential targets for cancer therapy [6, 7]. However, the role of most lncRNAs in cancer progression still remains unclear.

Plasmacytoma variant translocation 1 (PVT1), a long intergenic non-coding RNA encoded by the human oncogene PVT1, is located adjacent to the MYC locus on the well-known cancer-related region 8q24 [8–12]. Interestingly, PVT1 can increases MYC protein expression by regulating MYC protein stability [9, 13, 14], and in turn table MYC protein acts as a transcription factor to upregulate PVT1 expression [11, 15]. PVT1 has been reported to be upregulated in a variety of cancers, and plays an important role in cancer progression [16–19]. Accumulating evidences indicated that PVT1 can regulate many cancer-related cellular processes, including growth, proliferation, apoptosis, and differentiation, and it contributes to tumorigenesis by participating in DNA rearrangements, encoding microRNAs, and interacting with MYC [13, 15]. Recent studies indicated that down-regulation of PVT1 expression inhibited non-small cell lung cancer cells proliferation, migration and invasion through epigenetically regulating large tumor suppressor 2 (LATS2) expression [20, 21]. Wang et al reported that PVT1 promoted proliferation, stem cell-like properties and cell cycling of hepatocellular carcinoma cells by stabilizing nucleolar protein 2 (NOP2) protein [22]. In addition, Li et al found that PVT1 might act as a “sponge” to inhibit miR-152 in gastric cancer cells [23]. Based on these studies and owing to its functions, PVT1 may be a consequential biomarker of cancer, and also be one of the causal factors for tumorigenesis in general.

Recent clinical studies have demonstrated that increased PVT1 expression is correlated with poor survival in different types of cancers, including gastric cancer [24–29], colorectal cancer [30], lung cancer [20, 21, 31, 32], hepatocellular carcinoma [22, 33], bladder cancer [34], pancreatic cancer [35], cervical cancer [36, 37], ovarian cancer [38], esophageal cancer [39] and osteosarcoma [40]. Moreover, dysregulation of PVT1 expression is significantly correlated with certain clinicopathological characteristics of cancer. However, most studies reported the prognostic value of PVT1 expression are limited by sample size and discrete outcomes. Therefore, we conducted a systematic review and quantitative meta-analysis to investigate the prognostic value of PVT1 expression as a prognostic biomarker in various cancers.

## RESULTS

### Study selection and study characteristics

**Figure 1:**
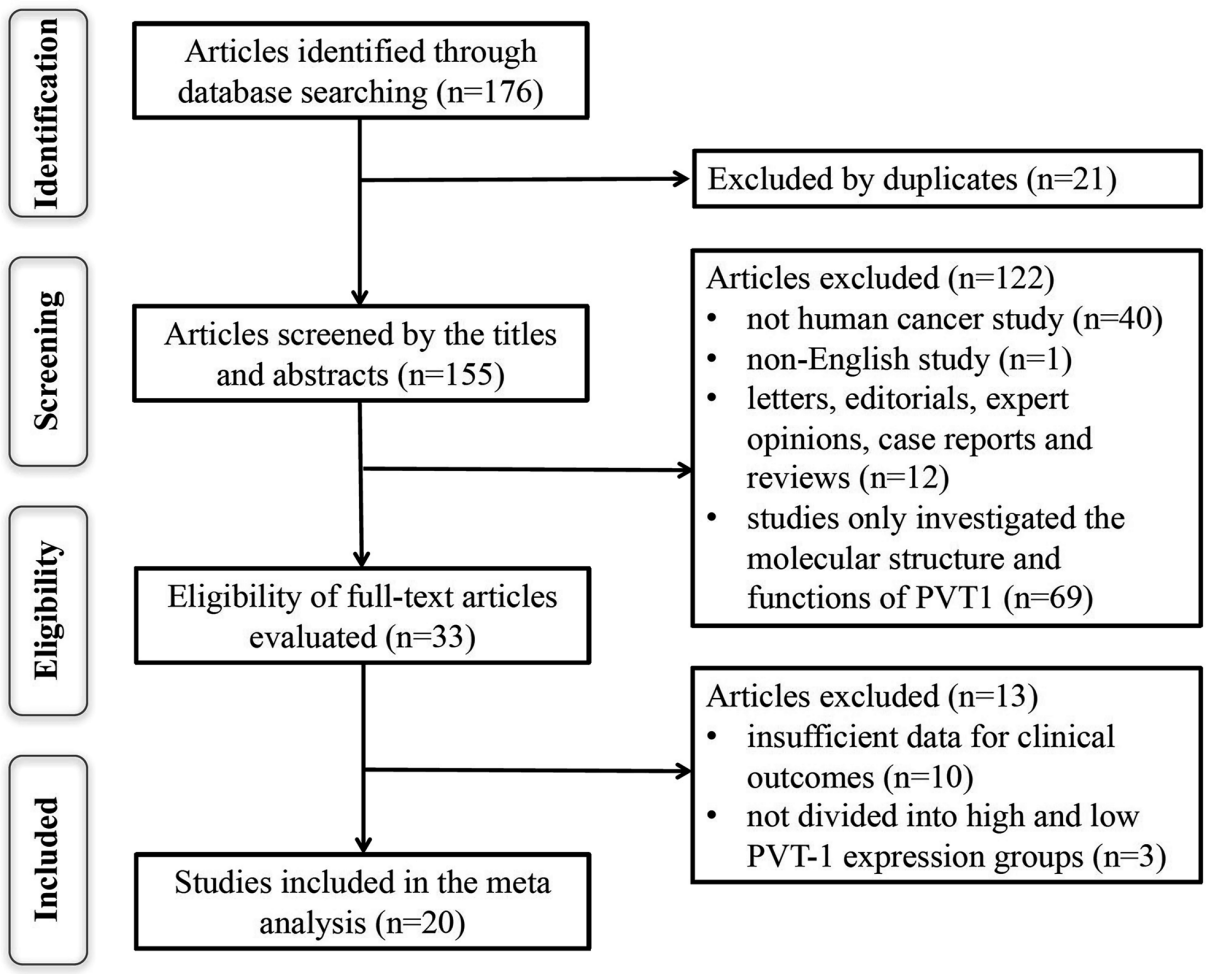
The flow diagram of this meta-analysis

A total of 176 potentially relevant articles were identified though the electronic search strategy. After removing duplications, 21 unique articles remained. Though careful the title and abstract screening, 122 irrelevant articles were excluded. After reviewing the full text of remaining 33 studies, 13 studies were excluded. Finally, 20 eligible studies were included in this meta-analysis. Flow diagram showing the selection of studies is present in Figure 1.

A total of 2109 patients from 20 studies between 2014 and 2017 were included. The regions of these studies included America (*n* = 1), Japan (*n* = 1), Italy (*n* = 1) and China (*n* = 17). Among all included studies, the sample size ranged between 26 and 214, and 10 studies enrolled more than 100. Eleven types of cancers were recorded including colorectal cancer (*n* = 1), hepatocellular carcinoma (*n* = 2), non-small cell lung cancer (*n* = 3), gastric cancer (*n* = 3), pancreatic ductal adenocarcinoma (*n* = 1), bladder cancer (*n* = 1), cervical cancer (n = 2), ovarian cancer (*n* = 1), esophageal cancer (*n* = 1) and small cell lung cancer (*n* = 1). The levels of PVT1 expression were detected by quantitative real-time polymerase chain reaction (qRT-PCR) in all included studies. The clinical outcomes were also recoded including 15 studies for OS, 2 for RFS, 5 for DFS, 2 for PFS, and 1 for DSS. HRs with the corresponding 95% CIs were extracted from the original data, including 9 studies for OS, 4 for DFS and 1 for PFS, and calculated from Kaplan-Meier curves in 6 studies for OS, 2 for RFS, 1 for DFS and 1 for PFS. The NOS scores of all included studies were more than 5. The characteristics of the included studies are summarized in Table 1.

**Table 1:** The main characteristics of the included studies in the meta-analysis

First author	Year	Region	Tumor Type	TNM Stage	Sample Size	Cut-off Value	Follow-up (months)	Detection Method	Adjuvant therapy before surgery	Survival Analysis		Outcome Measure		NOS
Takahashi Y	2014	Japan	CRC	0-IV	164	> 20%	45.6 (mean)	qRT-PCR	N/A	Univariate	Multivariate	OS		7
Wang F	2014	China	HCC	I-IV	89	Median	N/A	qRT-PCR	N/A	N/A		OS	RFS	7
Yang YR	2014	China	NSCLC	I-III	82	Median	41 (mean)	qRT-PCR	None	Univariate	Multivariate	OS		7
Ding J	2014	China	GC	I-IV	31	T/N>1	N/A	qRT-PCR	N/A	N/A		N/A		7
Ding CF	2015	China	HCC	I-IV	214	ROC	27.58 (mean)	qRT-PCR	N/A	N/A		OS	RFS	7
Kong R	2015	China	GC	I-IV	80	Median	N/A	qRT-PCR	N/A	Univariate	Multivariate	OS	DFS	7
Huang C	2015	China	PDAC	I-IV	85	Mean	10.2 (median)	qRT-PCR	None	Univariate	Multivariate	OS		7
Cui D	2015	China	NSCLC	I-IV	108	Median	32(median)	qRT-PCR	N/A	Univariate	Multivariate	OS	DFS	7
Zhuang CL	2015	China	BC	0-IV	32	N/A	N/A	qRT-PCR	N/A	N/A		N/A		7
Yuan C	2016	China	GC	I-IV	111	Median	36 (median)	qRT-PCR	N/A	Univariate	Multivariate	OS	DFS	7
Wan L	2016	China	NSCLC	I-IIIa	105	Median	N/A	qRT-PCR	None	Univariate	Multivariate	OS	PFS	7
Zhang SR	2016	China	CC	N/A	90	Median	60 (total)	qRT-PCR	N/A	N/A	N/A	OS		5
Martini P	2016	Italy	OC	I	129	Median	72(mean)	qRT-PCR	N/A	Univariate	Multivariate	OS	PFS	7
Xu MD	2016	China	GC	I-IV	190	Mean	32.43(mean)	qRT-PCR	None	Univariate	Multivariate	DFS	DSS	7
Zheng XX	2016	China	EC	I-IV	77	Median	N/A	qRT-PCR	N/A	N/A	N/A	N/A		7
Zhou Q	2016	China	Osteosarcoma	N/A	26	N/A	60 (total)	qRT-PCR	N/A	N/A	N/A	OS		5
Iden M	2016	America	CC	N/A	121	Median	60 (total)	qRT-PCR	N/A	N/A	N/A	OS		5
Huang CS	2016	China	SCLC	N/A	120	Median	96 (total)	qRT-PCR	None	Univariate	Multivariate	OS		7
Huang T	2017	China	GC	I-IV	68	Mean	N/A	qRT-PCR	N/A	N/A	N/A	N/A		7
Chen J	2017	China	GC	I-IV	187	N/A	26(median)	qRT-PCR	None	Univariate	Multivariate	OS	DFS	7

Abbreviations: CRC, colorectal cancer; HCC, hepatocellular carcinoma; NSCLC, non-small cell lung cancer; GC, gastric cancer; PDAC, pancreatic ductal adenocarcinoma; BC, bladder cancer; CC, cervical cancer; OC, ovarian cancer; EC, esophageal cancer; SCLC, small cell lung cancer; N/A, not available; T, tumor; N, normal; qRT-PCR, quantitative real-time polymerase chain reaction; OS, overall survival; DFS, disease-free survival; RFS, recurrence-free survival; PFS, progression-free survival; DSS, disease specific survival.

### Association between PVT1 expression and survival in different types of cancers

**Figure 2:**
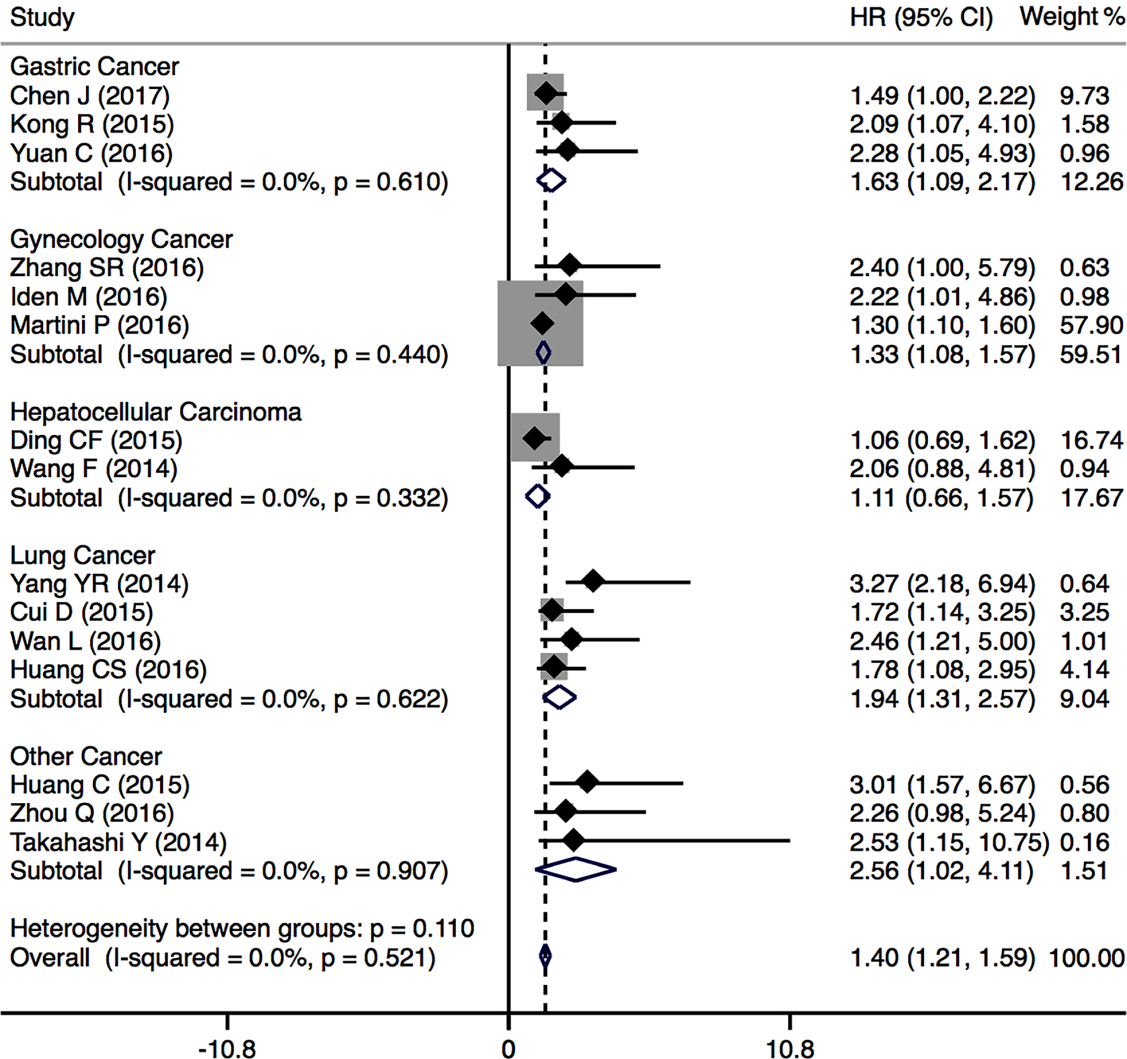
Forest plots of the included studies evaluating the HRs for PVT1 expression for OS by type of cancer

A total of 15 studies including 1711 patients were recruited to assess the effect of PVT1 expression on OS in various cancers. The results suggested that elevated PVT1 expression predicted a poor clinical outcome for OS in nine types of cancers (HR = 1.40, 95% CI: 1.21–1.59, *P* < 0.001) with no heterogeneity (I^2^ = 0.0%, *P* = 0.907). To maximize clinical relevance, subgroup analysis was conducted based on type of cancer, sample size, country and NOS scores. The results found that there was a significant relationship between PVT1 overexpression and poor OS of patients with gastric cancer (HR = 1.63, 95% CI: 1.09–2.17, *P* < 0.001), gynecology cancer (HR = 1.33, 95% CI: 1.08–1.57, *P* < 0.001) and lung cancer (HR = 1.94, 95% CI: 1.31–2.57, *P* < 0.001) (Figure 2). However, there was no significant association between PVT1 expression and OS in hepatocellular carcinoma. Furthermore, the negative effect of PVT1 overexpression on predicting poor OS was found in studies with sample size < 100 (HR = 2.40, 95% CI: 1.56–3.23) as well as those with sample size > 100 (HR = 1.35, 95% CI: 1.151.54) (Supplementary Figure 1). There was a significant relationship between PVT1 overexpression and poor OS in studies with NOS < 6 (HR = 2.28, 95% CI: 1.05–3.51) and NOS > 6 (HR = 1.38, 95% CI: 1.19–1.57) (Supplementary Figure 2). We also found a significant association between PVT1 overexpression and poor OS in Caucasian (HR = 1.32, 95% CI: 1.07–1.56) and Asian population (HR = 1.52, 95% CI: 1.23–1.82) (Supplementary Figure 3).

**Figure 3:**
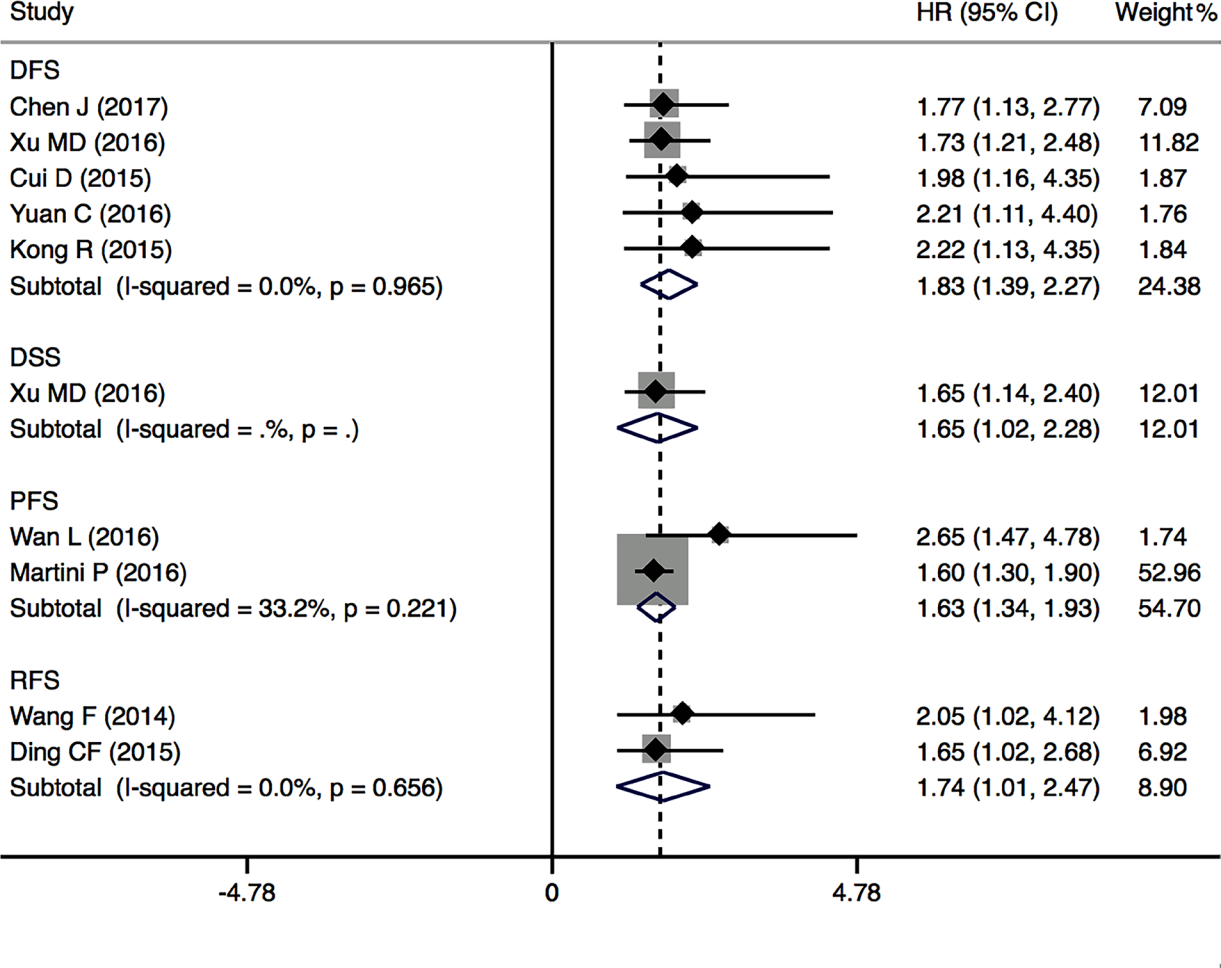
Forest plots of the included studies evaluating the HRs for PVT1 expression for DFS, RFS and PFS

In addition, the prognostic value of PVT1 expression in DFS was evaluated for 5 studies including 676 cancer patients. We found a negative significant relationship between high PVT1 expression and poor DFS (pooled HR = 1.83, 95% CI: 1.39–2.27) with no heterogeneity (*I*^2^ = 0.0%, *P* = 0.965) (Figure 3). Two studies including 234 cancer patients reported the HRs for PFS, and the results found that upregulation of PVT1 expression was associated with poor PFS (HR = 1.63, 95% CI: 1.34–1.93). Two studies comprising 403 cancer patients reported the HRs for RFS, and the results shown that PVT1 overexpression was significantly related with poor RFS (HR = 1.74, 95% CI: 1.01–2.47).

### Association between PVT1 expression and clinicopathological characteristics of cancers

**Figure 4:**
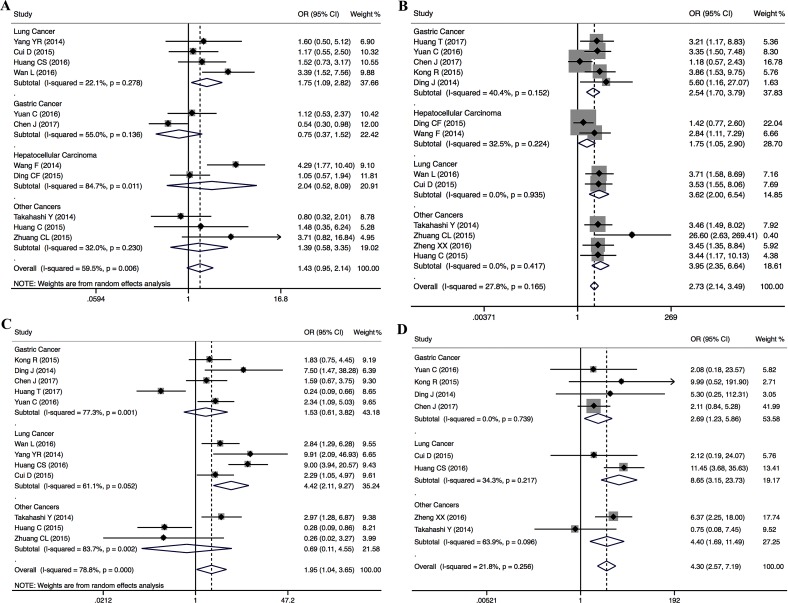
Subgroup analysis of the ORs for tumor size (**A**), TNM stage (**B**), lymph node metastasis (**C**) and distant metastases (**D**) by type of cancer.

To assess the association between PVT1 expression and clinicopathological characteristics of cancers, nine types of cancers from 15 studies were included. The results demonstrated that the level of PVT1 expression was significantly associated with tumor size (OR = 1.43, 95% CI: 0.95–2.14, *P* = 0.006), TNM stage (OR = 2.73, 95% CI: 2.14–3.49, *P* < 0.001), lymph node metastasis (OR = 1.95, 95% CI: 1.04–3.65, *P* = 0.037), or distant metastases (OR = 4.30, 95% CI: 2.57–7.19, *P* < 0.001) (Table 2). However, there was no significant association between PVT1 expression and age, gender or differentiation. Furthermore, subgroup analysis was conducted based on type of cancer and sample size. As indicated in Figure 4A, 1297 patients from 11 studies examined the association between tumor size and PVT1 expression in different types of cancers, including lung cancer (*n* = 4), gastric cancer (*n* = 2), hepatocellular carcinoma (*n* = 2), colorectal cancer (*n* = 1), pancreatic cancer (*n* = 1) and bladder cancer (*n* = 1). The results shown that there was a significant association between tumor size and PVT1 expression in lung cancer (OR = 1.75, 95% CI: 1.17–2.64) and hepatocellular carcinoma (OR = 1.67, 95% CI: 1.02–2.73). In particular, the results also found that PVT1 was a safe prognostic factor for gastric cancer (OR = 0.72, 95% CI: 0.45–1.14). As shown in Figure 4B, 13 studies including 1351 patients were enrolled to evaluate the association between TMN stage and PVT1 expression in different cancers, such as gastric cancer (*n* = 5), hepatocellular carcinoma (*n* = 2), lung cancer (*n* = 2), colorectal cancer (*n* = 1), pancreatic cancer (*n* = 1), esophageal cancer (*n* = 1) and bladder cancer (*n* = 1). The results found a significant relationship between TMN stage and PVT1 expression in gastric cancer (OR = 2.54, 95% CI: 1.70–3.79), hepatocellular carcinoma (OR = 1.75, 95% CI 1.05–2.90) and lung cancer (OR = 3.62, 95% CI 2.00–6.54). Furthermore, 12 studies comprising 1173 patients were pooled to evaluate the association between lymph node metastases and PVT1 expression in different cancers, such as gastric cancer (*n* = 5), lung cancer (*n* = 4), colorectal cancer (*n* = 1), pancreatic cancer (*n* = 1) and bladder cancer (*n* = 1). Stratified analysis by type of cancer indicated a significant relationship between PVT1 overexpression and high incidence of lymph node metastasis in lung cancer (OR = 4.42, 95% CI 2.11–9.27) (Figure 4C). Moreover, the negative effect of PVT1 overexpression on predicting high incidence of lymph node metastasis was shown in studies with > 100 patients (pooled OR = 2.93, 95% CI: 1.85–4.66) (Supplementary Figure 4). In the present study, we also evaluated the association between distant metastases and PVT1 expression for 8 studies including 878 patients with four different cancers, including gastric cancer (*n* = 4), lung cancer (*n* = 2), colorectal cancer (*n* = 1) and esophageal cancer (*n* = 1). A significant association was found between distant metastases and PVT1 in gastric cancer (OR = 2.69, 95%CI: 1.23–5.86) and lung cancer (OR = 8.65, 95% CI 3.15–23.73) (Figure 4D).

**Table 2:** Correlation between PVT1 expression and clinicopathological characteristics of cancers

Clinical parameters	No. of studies	No. of patients	OR (95% CI)	*P*-value	Heterogeneity	
					*I*^2^	*P*-value
Age (elderly vs. young)	14	1522	0.86 (0.69–1.08)	0.200	0%	0.940
Gender (male vs. female)	15	1553	1.05 (0.83–1.31)	0.680	0%	0.543
Tumor size (large vs. small)	11	1297	1.43 (0.95–2.14)	0.006	59.5%	0.084
Differentiation (poor vs. well)	10	1131	1.22 (0.76–1.95)	0.416	55.2%	0.017
TNM stage (III + IV vs. I + II)	13	1351	2.73 (2.14–3.49)	< 0.001	27.8%	0.165
Lymph node metastasis (present vs. absent)	12	1173	1.95 (1.04–3.65)	0.037	78.8%	< 0.001
Distant metastasis (present vs. absent)	8	878	4.30 (2.57–7.19)	< 0.001	21.8%	0.256

### Publication bias

**Figure 5:**
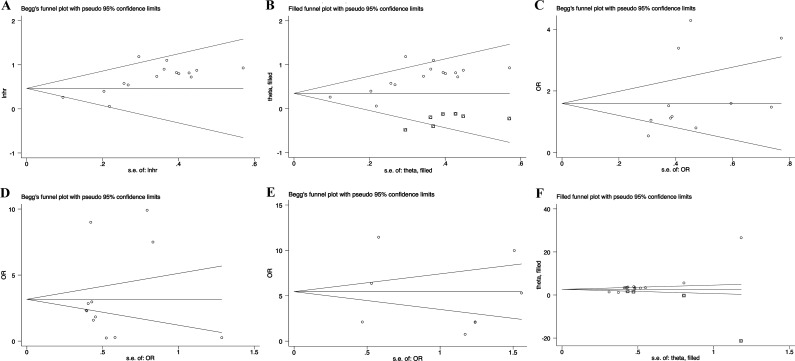
Funnel plot and trim and fill analysis evaluating publication bias (**A**) Begg funnel plot with pseudo 95% CIs for OS; (**B**) Filled funnel plot with pseudo 95% CIs for OS; (**C**) Begg funnel plot with pseudo 95% CIs for tumor size; (**D**) Begg funnel plot with pseudo 95% CIs for TNM stages; (**E**) Begg funnel plot with pseudo 95% CIs for lymph node metastasis; (**F**) Filled funnel plot with pseudo 95% CIs for distant metastases.

To evaluate the publication bias, the funnel plot and Begg's test were used. In the analysis of evaluating the association between PVT1 expression on overall survival, visual inspection of the Begg's funnel plot revealed asymmetry (Figure 5A), Begg's test suggested the probable evidence of publication bias (*t* = 4.68, *P* < 0.001). To assess the impact of potential publication bias, the trim and fill analysis was performed with the fixed-effects model. Seven which conservatively imputes hypothetical negative unpublished studies to mirror the positive studies that cause funnel plot asymmetry. The imputed studies produce a symmetrical funnel plot (Figure 5B). The pooled analysis incorporation the hypothetical studies continued to show a statistically significant association between PVT1 expression on OS (corrected HR = 1.42, 95% CI: 1.26–1.59, *P* < 0.001). Due to the small number of studies, the publication bias was not analyzed in the DFS, DSS, RFS and PFS groups.

In addition, we also evaluated the association between PVT1 expression and lymph node metastasis, tumor size or distant metastases. Visual inspection of the funnel plot and Begg's test suggested that there was no evidence of publication bias (lymph node metastasis (*t* = 0.43, *P* = 0.675), tumor size (*t* = 1.45, *P* = 0.180) or distant metastases (*t* = -0.22, *P* = 0.832), respectively) (Figure 5C–5E). However, the funnel plot showed obvious asymmetry among the studies investigating PVT1 expression on TNM stage (*t* = 5.16, *P* < 0.001). The corrected OR regarding the association between PVT1 expression and TNM stages was 2.63 (95% CI: 2.40–2.86, *P* < 0.001), which showed that the correction for potential publication bias had an influence on the stability of the result (Figure 5F).

## DISCUSSION

Cancer has already become one of the major public health issues in most regions of the world. Despite current advances in cancer treatment, patients with various cancers have still comparatively poor prognosis [41]. An increasing number of studies have focused on the involvement of lncRNAs in cancer development and progression [17, 42–44]. Especially, PVT1, regarded as one of the most familiar oncogenic lncRNAs, exhibited the ability to improve the development and progression of different cancers [16, 17, 45, 46]. In recent years, numerous studies have demonstrated that high PVT1 expression significantly correlated with poor prognosis of patients with nearly all types of cancers [25, 26, 30, 31, 33, 35]. However, most studies reported the prognostic value of PVT1 expression are limited by sample size and discrete outcomes [33]. As a result, it is important to combine these published data by meta-analysis and evaluate the association between PVT1 expression and prognosis as well as clinicopathological characteristics in cancer patients.

In order to clarify the association between PVT1 expression and clinical outcomes in cancer patients, we conducted a meta-analysis of 15 studies including 1711 patients to assess the effect of PVT1 expression on overall survival of patients with various cancers. Our results suggested that elevated PVT1 expression predicted a poor clinical outcome for overall survival in nine types of cancers (HR = 1.40, 95% CI: 1.21–1.59). Due to large difference of the clinical relevance among various types of cancers, we also performed the subgroup analysis by types of cancer. And those results found that there was a significant relationship between PVT1 overexpression and poor overall survival of patients with gastric cancer, gynecology cancer and lung cancer. However, no significant association was found between PVT1 expression and overall survival of HCC patients. The key reason of this negative result may be attributed to Ding's study [33]. Although they found no statistically significance of PVT1 as a predictor of HCC patients’ survival, HCC patients with high level of PVT1 expression demonstrated a trend for poor prognosis [33]. Other research group demonstrated that PVT1 promotes cell proliferation, cell cycling, and the acquisition of stem cell-like properties in HCC cells by stabilizing NOP2 protein, and HCC patients with high PVT1 expression had a poor prognosis [22]. Furthermore, our data suggested the probable evidence of publication bias among the studies investigating the effects of PVT1 expression on overall survival. The trim and fill analysis showed that seven which conservatively imputes hypothetical negative unpublished studies to mirror the positive studies that cause funnel plot asymmetry. The pooled analysis incorporation the hypothetical studies continued to show a statistically significant association between PVT1 expression on overall survival, and this results indicated that the correction for potential publication bias had an effect on the stability of the results. As a result, these findings indicated that PVT1 may act as a promising prognostic biomarker to predict patients’ survival in different types of cancers.

Accumulated evidence suggested that PVT1 overexpression was associated with high-risk grade, metastasis, and poor overall survival of cancer patients, and acted as a powerful predictor of tumor progression in a wide range of cancers. Many studies have suggested using the levels of PVT1 expression to predict lymph node metastasis in gastric cancer [24], histological grade and lymph node metastasis in non-small cell lung cancer [31, 32], clinical stage and N-classification in pancreatic cancer [35], and the recurrence rate in HCC [33]. PVT1 have been reported to function in multiple biological processes associated with cancer progression, including proliferation, apoptosis and invasion [47–49]. However, the precise mechanism of how PVT1 promotes tumor progression is unclear. Xu et al. found that PVT1 could facilitate gastric cancer growth and invasion by interacting with forkhead box M1(FOXM1). Some studies suggested that overexpression of PVT1 exhibited the anti-apoptotic property and promoted the development of multidrug resistance in gastric cancer and ovarian cancer [18, 50]. Other study reported that PVT1 promotes cell proliferation through epigenetically regulating p15 and p16, and higher PVT1 expression in gastric cancer was positively correlated with deeper invasion depth and advanced TNM stage [26]. In addition, Zheng et al. found that upregulation of PVT1 expression may induce epithelialtomesenchymal transition (EMT) to promote cell invasion in esophageal cancer by regulating the expression levels of Ecadherin, Ncadherin and vimentin [39].

In this study, we also assessed the association between PVT1 expression and clinicopathological characteristics in nine types of cancers. We found that there was a significant association between tumor size and PVT1 expression in lung cancer and HCC. Although PVT1 overexpression acted as a safe prognostic factor for tumor size of gastric cancer, it predicted the high incidence of distant metastases in gastric cancer. Our results also indicated that increased PVT1 expression significantly related with TMN stages in gastric cancer, HCC and lung cancer, and predicted high incidence of lymph node metastasis and distant metastases in lung cancer. Furthermore, no evidence of publication bias was found in the studies investigating the association between PVT1 and tumor size, lymph node metastasis or distant metastases. However, the funnel plot showed obvious asymmetry among the studies investigating the association between PVT1 and TNM stage. the trim and fill analysis showed that the correction for potential publication bias had an influence on the stability of the results.

Limitations of this meta-analysis must be considered due to the discrete data across these clinical studies. Firstly, the quality of individual studies was not always optimal, as shown by the general lack of information on blinding and recruiting of consecutive patients for all studies. Secondly, if the HRs of overall survival was not reported, it would be calculated from the data included in the articles or extrapolated from the Kaplan-Meier survival curves. In fact, the method of calculating the HRs from the survival curves was less reliable than directly extracting from the primary studies, thus a calculation bias might be present. Thirdly, in all included studies, the criteria for calculating the cut-off value were not the same in different studies. The inclusion of a relatively small number of studies in different regions (China, America, Italy and Japan) might have decreased the applicability of our results across different ethnicities. The data collection may be incomplete because data from non-English language papers were not included. Finally, the funnel plot analysis showed some asymmetry, suggesting the possibility of publication bias in OS and TMN stage groups. However, the trim and fill sensitivity analysis did not change the general result, suggesting that the bias possibly resulted from unpublished negative studies. Although the publication bias existed, sensitivity analyses demonstrated the reliability of our meta-analysis.

In conclusion, this meta-analysis systematically evaluated the association between PVT1 expression and prognosis as well as clinicopathological characteristics in cancer patients. Our findings indicated that the increased PVT1 expression not only predicted poor prognosis of cancer patients, but also positively correlated with tumor size, TNM stage, lymph node metastasis, and distant metastases. Therefore, PVT1 may potentially be used as a novel biomarker for predicting patients’ prognosis in different types of cancers. More clinical studies on other different types of human cancers that have not yet been investigated needed to be conducted.

## MATERIALS AND METHODS

### Search strategy and study selection

An electronic search of the PubMed, EMBASE, Web of Science, Ovid and Cochrane library databases were undertaken for eligible studies evaluating the prognostic value of PVT1 expression in cancers. The following search terms were included: “PVT1”; “plasmacytoma variant translocation 1”; “long intergenic noncoding RNA” or “lncRNA” or “noncoding RNA”; “cancer” or “carcinoma” or “neoplasm”; and “prognosis” or “survival”. The literature search was limited to the English language and ended in June, 2017.

A total of 176 potentially relevant articles were identified. To yield relevant articles, we further evaluated the titles, abstract and author information of the collected studies. For the analysis and full text review, non-research articles such as letters, editorials, expert opinions, case reports, reviews and other type uninvolved publications were excluded. For evaluated the eligibility of full-text articles, the articles without the sufficient data or dividing into high and low PVT1 expression groups were excluded. The studies were considered eligible if they met the following inclusion criteria: (i) any type of human cancer was involved; (ii) studies evaluating the prognostic value of PVT1 in cancers; (iii) PVT1 expression in human tissues were detected; (iv) studies evaluating the relationship between PVT1 and the clinicopathologic parameters was included; (v) studies providing the survival curve or sufficient data to estimate the HRs with 95% CI for OS, DFS, PFS or RFS; and (vi) studies were published in English. For this meta-analysis, the studies were excluded if they met the following exclusion criteria: (i) duplicate publications, (ii) letters, editorials, expert opinions, case reports and reviews; (iii) studies only investigating the molecular structure and functions of PVT1; (iv) studies without the survival curve or sufficient data to be extracted or calculated from the original article; and (v) studies without dividing into high and low PVT1 expression groups. Two investigators (Shikai Zhu and Yu Zhou) independently evaluated and extracted the data from each eligible study. A consensus was achieved for disagreements by a third investigator (Hong-Ji Yang).

### Data extraction and quality assessment

Data extraction was assessed independently by three investigators (Shikai Zhu, Yu Zhou and Chong Yang), and a consensus was reached for each data set of the included articles. According to the above inclusion and exclusion criteria, the following items were extracted: first author, publication date, country, ethnicity, tumor type, sample size, detection method of PVT1 expression, follow-up period, cut-off value, the number of high and low PVT1 expression, the number of patients with lymph node metastasis, adjuvant therapy before surgery, survival analysis methodology, HRs with corresponding 95% CIs for OS, RFS or DFS, and other patient data such as age, gentle, tumor size, differentiation, TNM staging and distant metastasis. HRs with corresponding 95% CIs were preferentially directly extracted from univariate or multivariate analyses in the original articles. If these were not available, the HR estimates were calculated from Kaplan-Meier survival curves using Engauge Digitizer V4.1 software according to the method of Tierney et al. [51].

The quality assessment of all included studies was performed based on the Newcastle-Ottawa quality assessment scale (NOS) [52]. The NOS criteria apply a “star” rating system ranges from 0 to 9 stars for the judgment of methodological quality based on selection (0–4 stars), comparability (0–2 stars) and outcome (0–3 stars). Based on previous recommendations, studies with 5 points were considered to be of high quality. The two aforementioned investigators evaluated each paper independently and then compared the results. Conflicting evaluations or inconsistent data from all included studies were resolved with a third investigator (Hongji Yang) vis discussion. The final NOS scores of the included studies are shown in Table 1.

### Statistical analyses

All data analyses were performed with STATA statistical software version 14.0 (Stata Corporation, College Station, TX, USA). Pooled HRs with 95% CIs were used to estimate the prognostic role of PVT1 in various cancers. Pooled ORs with 95% CIs were used to estimate the association between PVT1 expression and clinicopathological characteristics. *X*^2^-based Cochran Q test and Higgins *I*^2^ statistic were utilized to determine the heterogeneity among the included studies. A *P*-value < 0.05 in combination with *I*^2^ value > 50% was considered significant heterogeneity. Random-effects models were applied in cases with significant heterogeneity. Subgroup analysis and sensitivity analysis were performed to dissect the heterogeneity. In addition, publication bias was determined using funnel plot and Begg's test. A *P*-value < 0.05 was considered statistically significant.

## SUPPLEMENTARY MATERIALS FIGURES


