# Supplementary material for: Prognostic value of long non-coding RNA PVT1 as a novel biomarker in various cancers: a meta-analysis
Source: Oncotarget. 2017 Dec 1;8(68):113174–84. doi: 10.18632/oncotarget.22830 (PMC5762581; doi:10.18632/oncotarget.22830)
Supplement: Supplementary file 1 [file oncotarget-08-113174-s001.pdf]

Prognostic value of long non-coding RNA PVT1 as a novel biomarker in various cancers: a meta-analysis

SUPPLEMENTARY MATERIALS

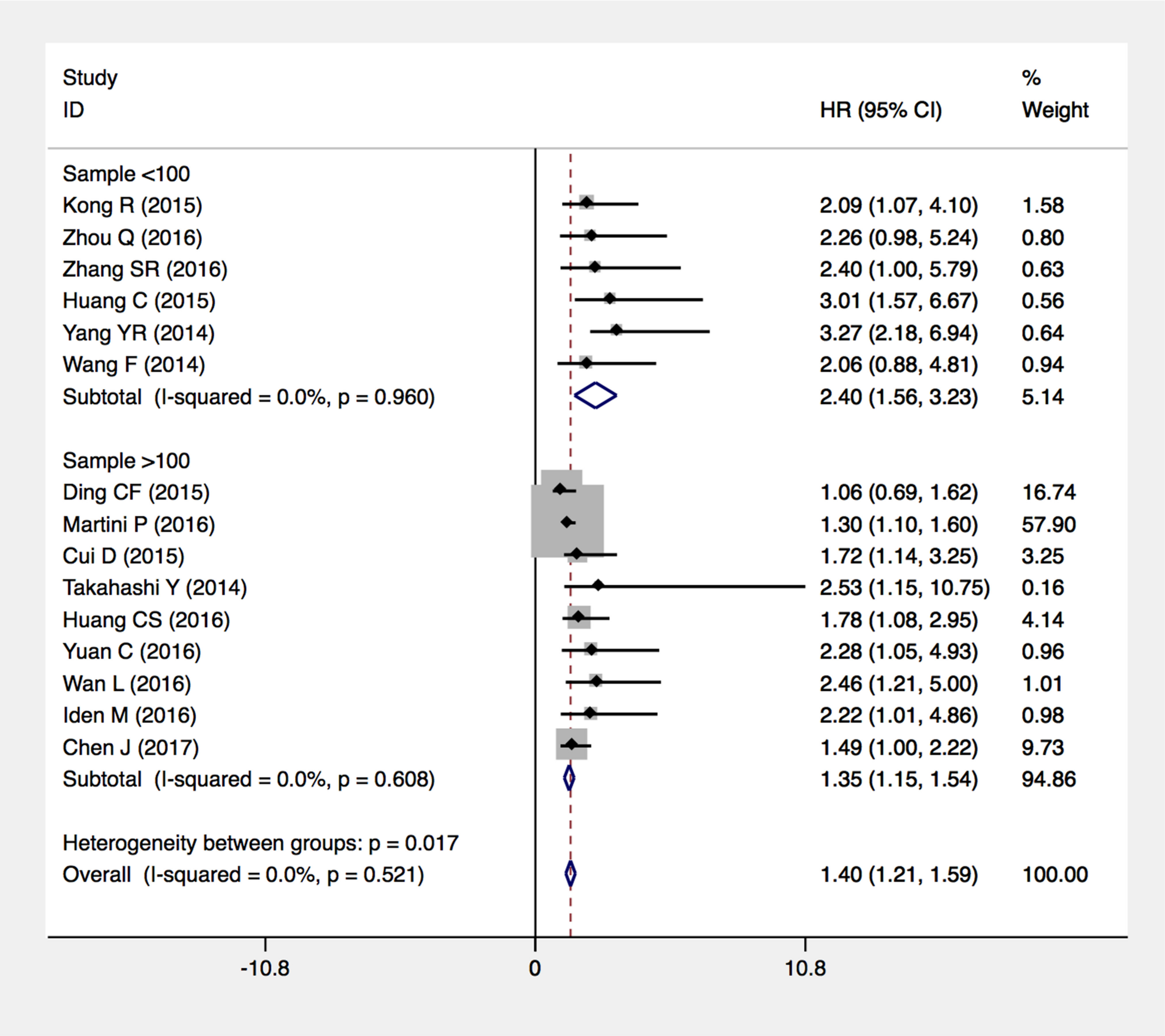

Supplementary Figure 1: Forest plots of the included studies evaluating the HRs for PVT1 expression for OS by sample size.

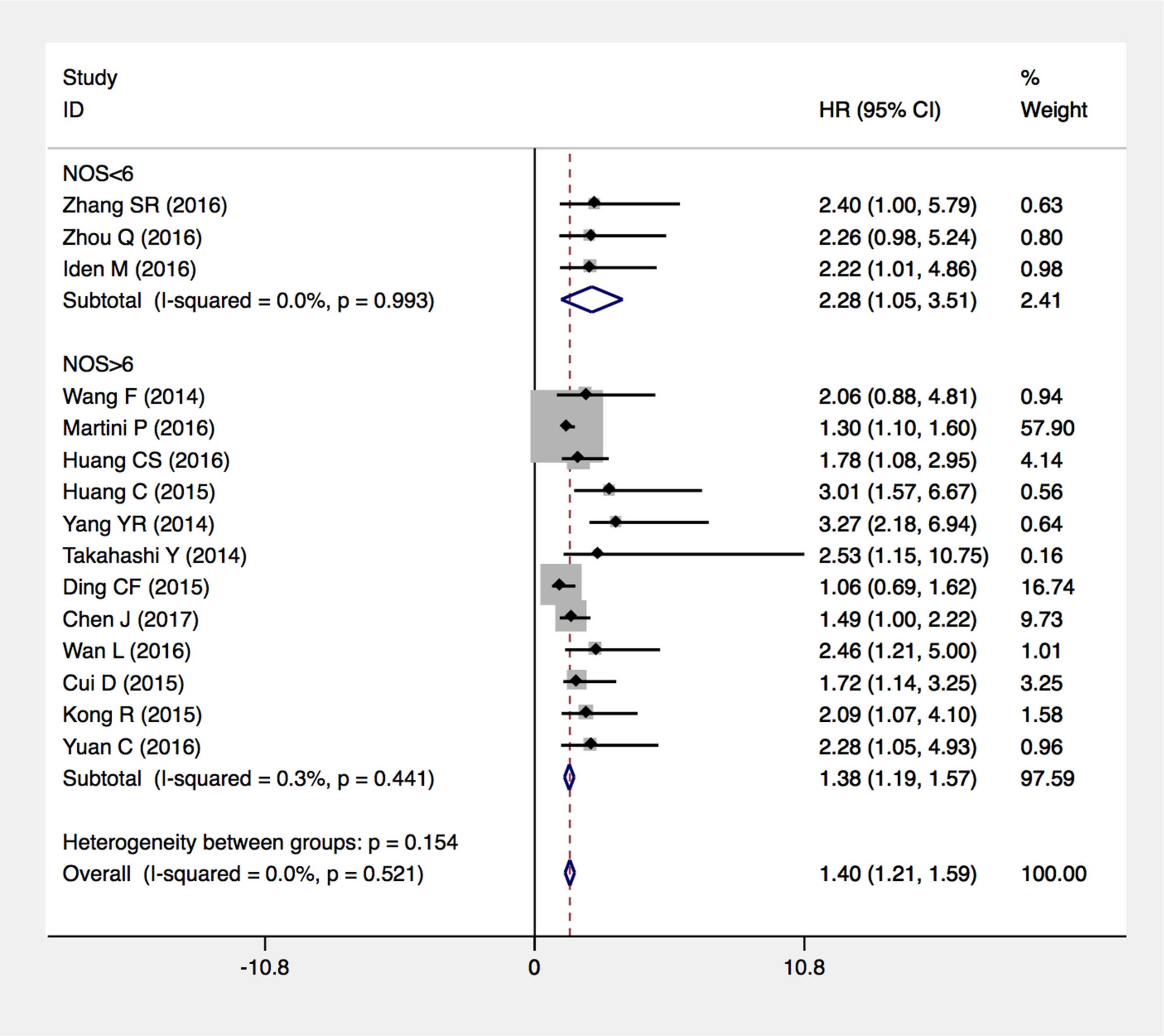

Supplementary Figure 2: Forest plots of the included studies evaluating the HRs for PVT1 expression for OS by NOS scores.

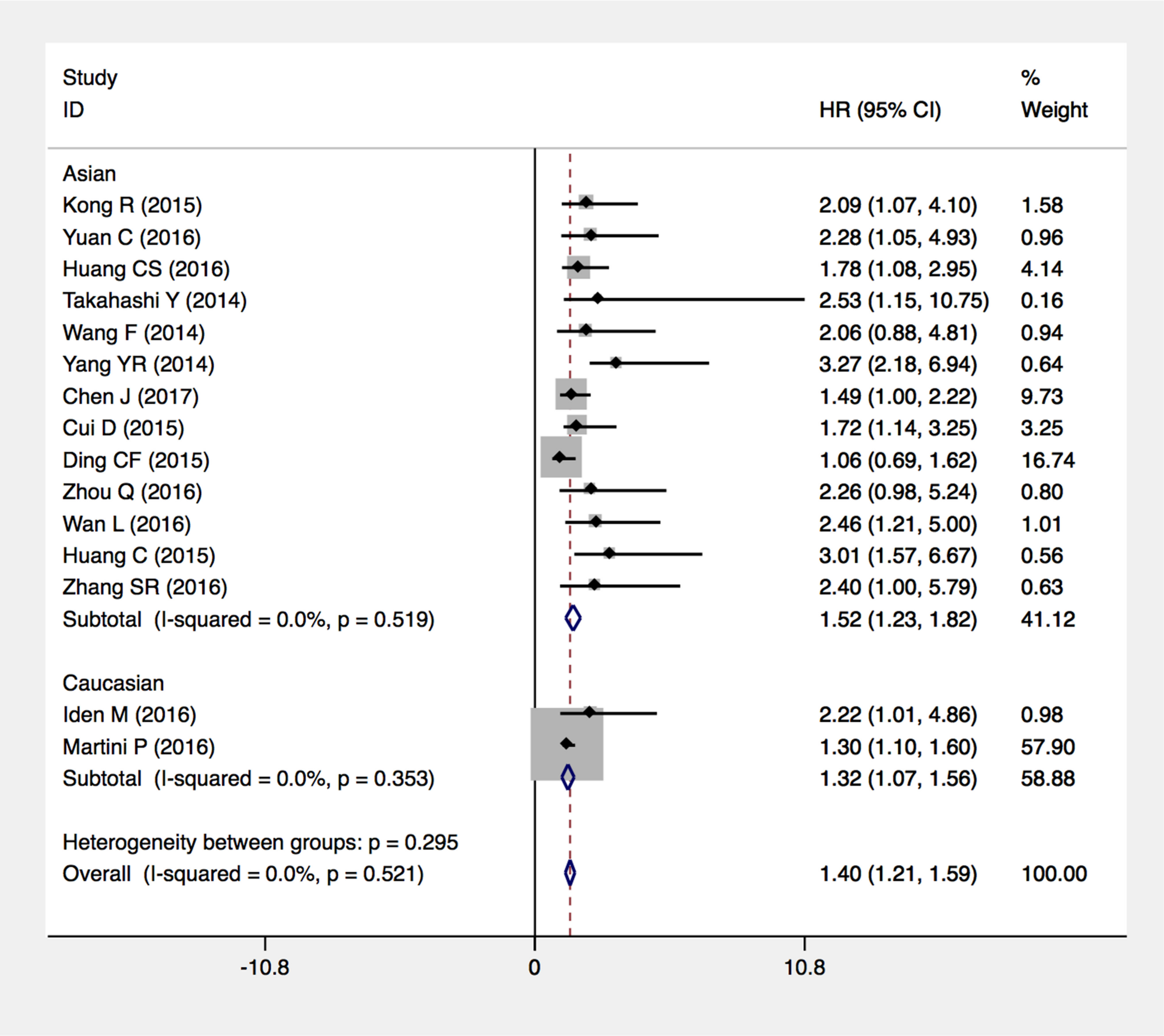

Supplementary Figure 3: Forest plots of the included studies evaluating the HRs for PVT1 expression for OS by country.

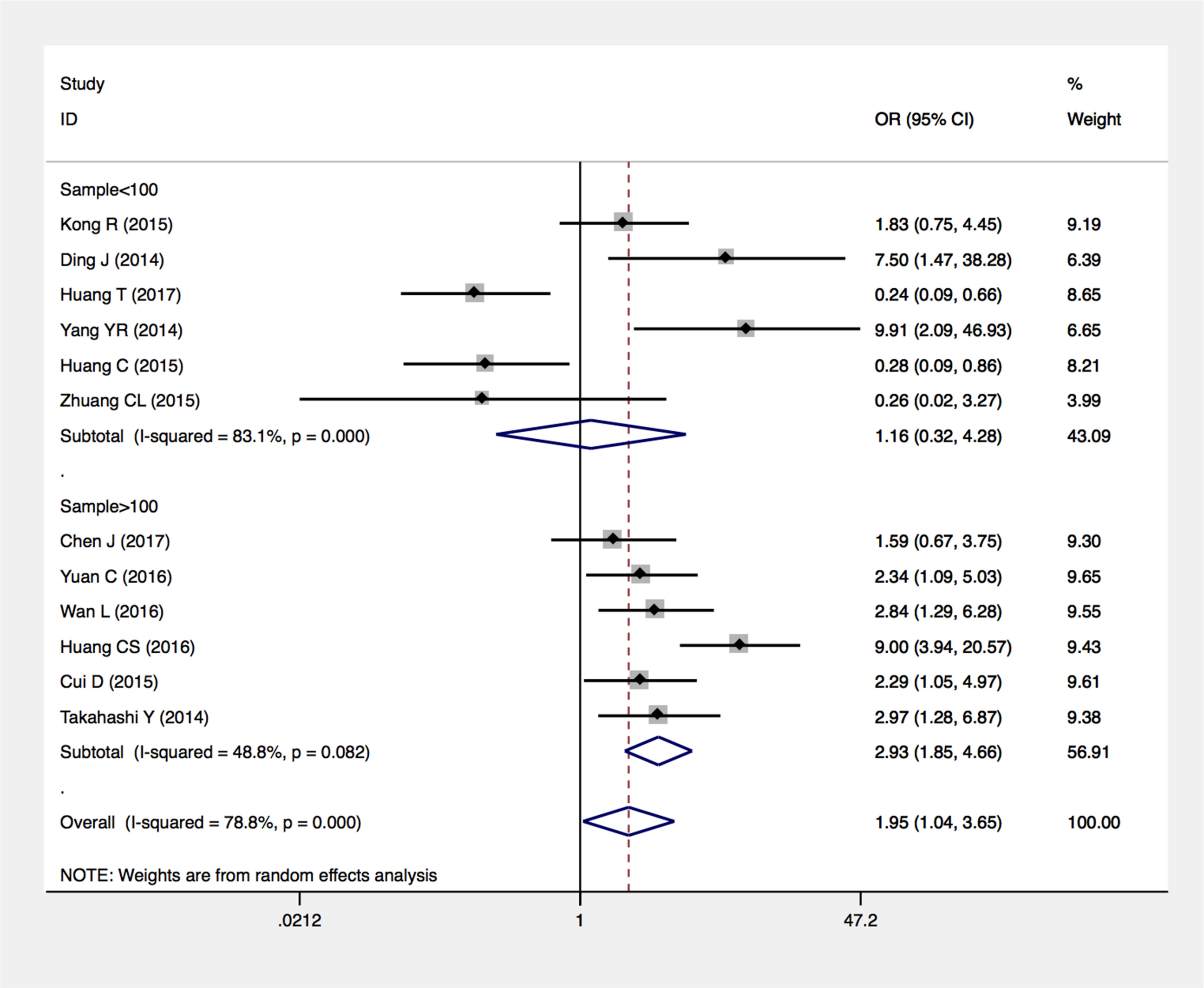

Supplementary Figure 4: Subgroup analysis of the ORs for lymph node metastasis by sample size.
